# Supplementary material for: Light control of the peptide-loading complex synchronizes antigen translocation and MHC I trafficking
Source: Commun Biol. 2021 Mar 30;4:430. doi: 10.1038/s42003-021-01890-z (PMC8010092; doi:10.1038/s42003-021-01890-z)
Supplement: Supplementary file 2 — Supplementary Information [file 42003_2021_1890_MOESM2_ESM.pdf]

Supplementary Figures

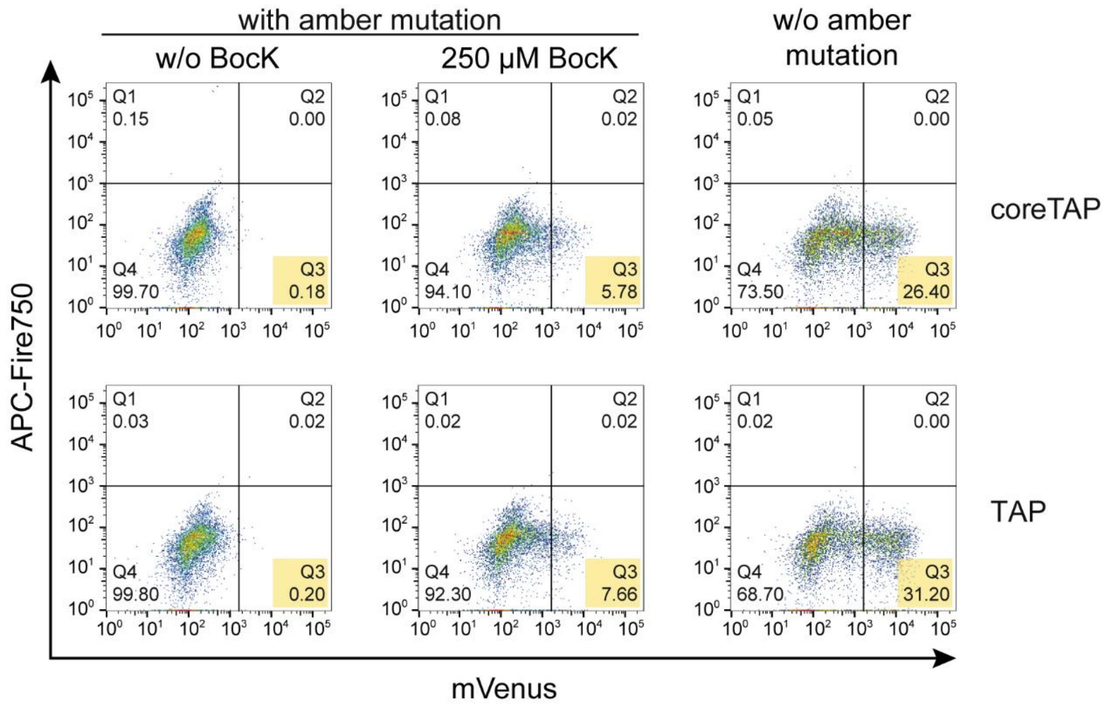

Supplementary Fig. 1 | Expression of TAP variants in TAP2-negative STF1-169 cells. Co-expression of amber-free and amber-suppressed coreTAP or TAP with wtPylRS in the presence and absence of 250 µM BocK (representative data, n=3, biologically independent samples).

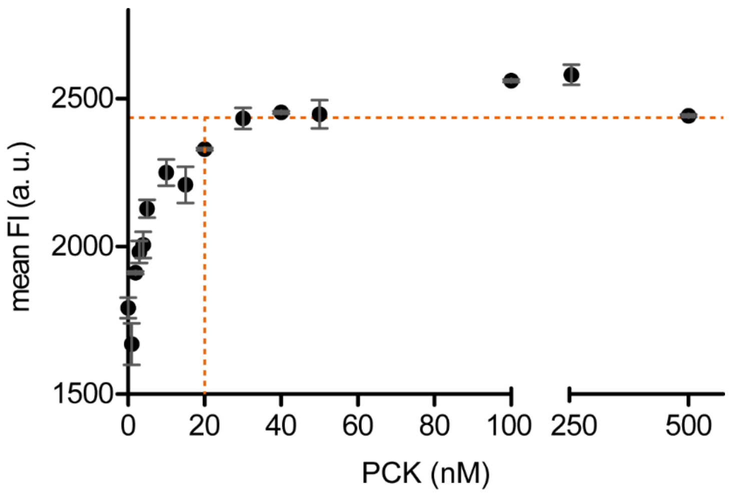

Supplementary Fig. 2 | PCK concentration required for amber suppression. TAP2-deficient STF1-169 cells, co-expressing coreTAP^{TAG} and optPylRS in the presence of varying PCK concentrations, were analyzed by flow cytometry. Mean FI of the mVenus positive cells (± SEM, n=3, biologically independent samples) are shown. FI, fluorescence intensity.

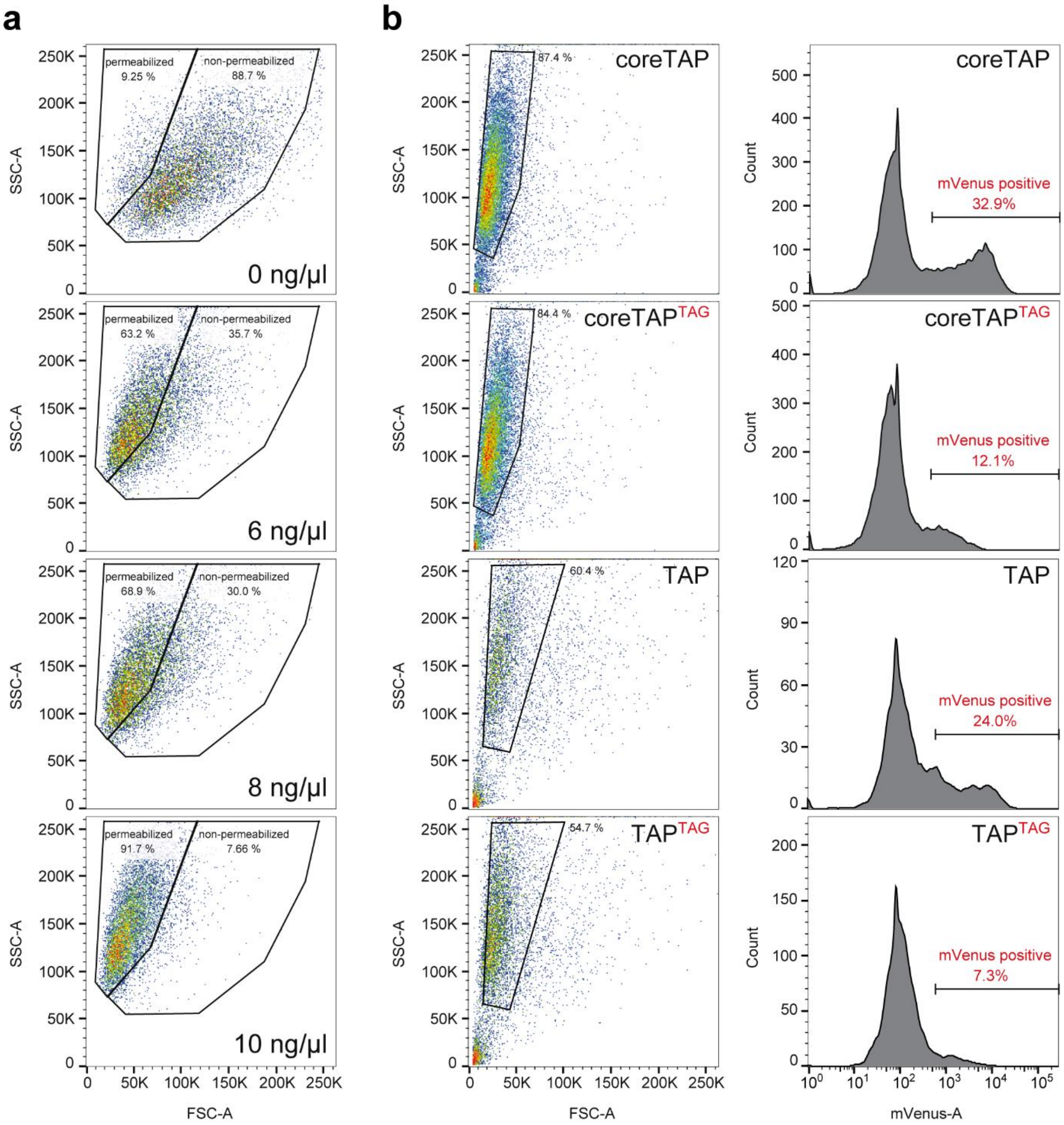

Supplementary Fig. 3 | Semi-permeabilization of STF1-169 cells and gating for peptide translocation assay. a, Untransfected TAP2-deficient STF1-169 cells were semi-permeabilized using 6, 8, or 10 ng/µl SLO (1 ng ≙ 0.55 U). Gating strategy for semi-permeabilized cells was previously described[48]. **b**, Amber-free and amber-suppressed coreTAP/PCK or TAP/PCK were expressed in STF1-169 cells. Cells were semi-permeabilized with 10 ng/µl SLO and utilized for the peptide translocation assay (representative data, n=3, biologically independent samples).

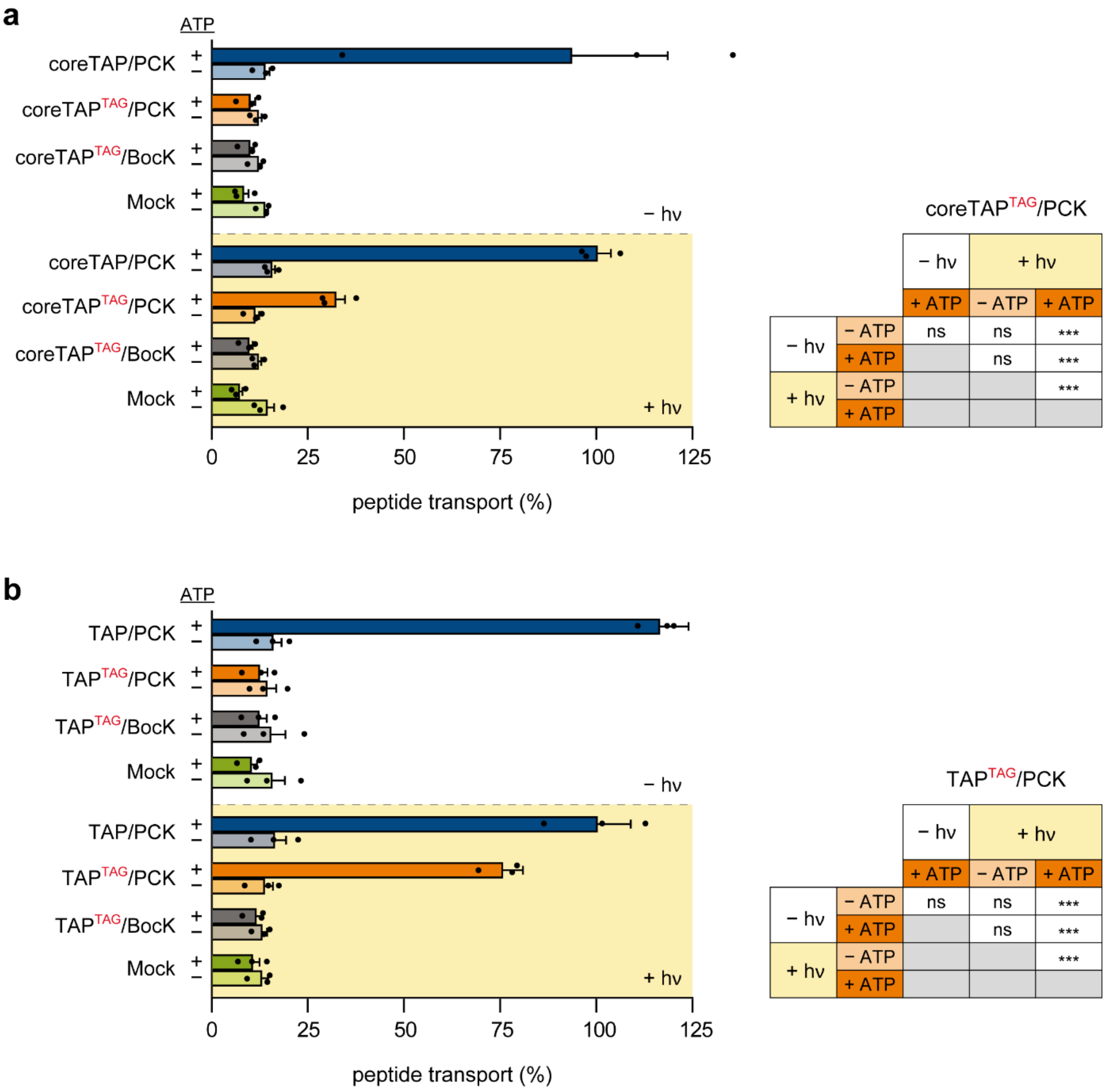

Supplementary Fig. 4 | Peptide translocation and statistics. a, Amber-free and amber-suppressed coreTAP and **b,** TAP variants were expressed in TAP2-deficient STF1-169 cells. Peptide translocation without illumination (– hv) and after light activation (+ hv, yellow shaded) was monitored by flow cytometry. Peptide transport was determined by normalizing the mean FI (± SEM, n=3, biologically independent samples) of transported peptide to the illuminated corresponding amber-free variant in presence of ATP. The ADP-samples are depicted in light colors, the ATP-samples in dark colors. One-way ANOVA with Turkey´s multiple comparison test was performed for photo-conditional coreTAP^{TAG} and TAP^{TAG}. ns, non-significant; ***, $p < 0.0001$. FI, fluorescence intensity.

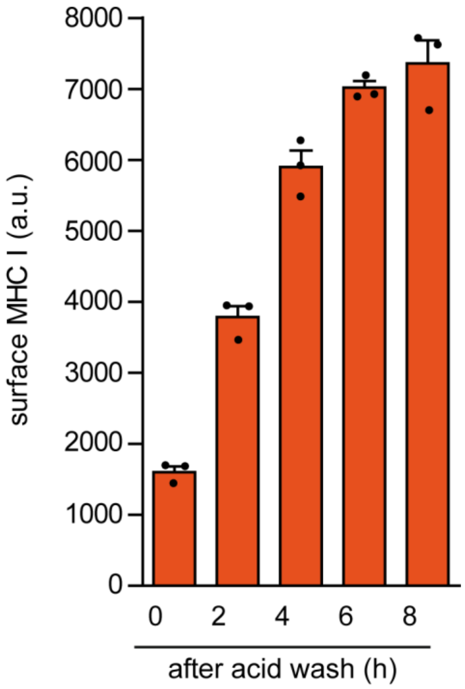

Supplementary Fig. 5 | Time-dependent recovery of MHC I at the cell surface. CoreTAP was expressed in TAP2-deficient STF1-169 cells. After acid wash, MHC I surface presentation was measured by flow cytometry using an APC-Fire750-labeled HLA-A, B, C-specific antibody (W6/32) and the mean FI (± SEM, n=3, biologically independent samples) of the mVenus positive cells were calculated. FI, fluorescence intensity.

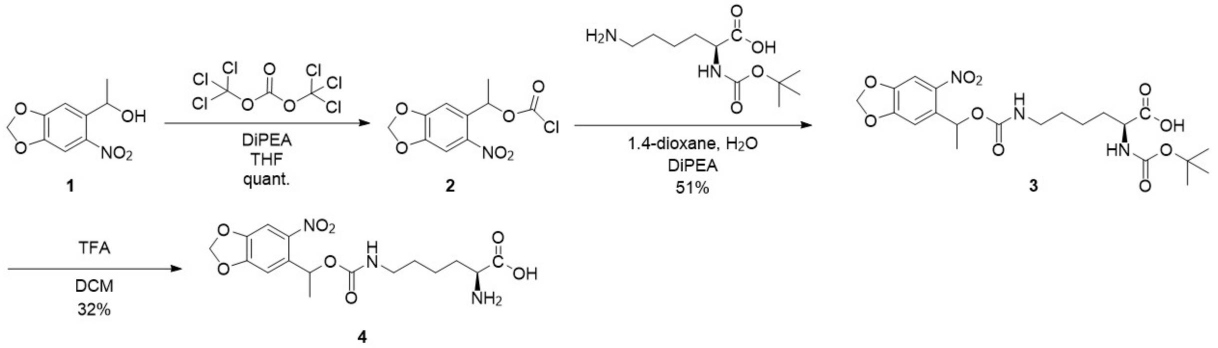

Supplementary Fig. 6 | Synthesis of nitropiperonyl caged lysine (PCK). 1-(6-Nitrobenzo[d][1,3]dioxol-5-yl)ethanol **1** was quantitatively converted to nitropiperonyl chloroformate **2** using triphosgene ((bis(trichloromethyl) carbonate). N_{ε}-Boc-lysine was added dropwise to the chloroformate **2** in order to yield compound **3**, which was finally deprotected by TFA to yield 2-amino-6-([1-(6-nitrobenzo[d][1,3]dioxol-5-yl)ethoxy]carbonylamino)hexanoic acid **4**.
