# Supplementary material for: Light control of the peptide-loading complex synchronizes antigen translocation and MHC I trafficking
Source: Commun Biol. 2021 Mar 30;4:430. doi: 10.1038/s42003-021-01890-z (PMC8010092; doi:10.1038/s42003-021-01890-z)
Supplement: Supplementary file 3 — Description of Additional Supplementary Files [file 42003_2021_1890_MOESM3_ESM.pdf]

## Description of Additional Supplementary Files

**File Name:** Supplementary Data 1

**Description:** Source data underlying the graphs.

**File Name:** Supplementary Data 2

**Description:** Source data underlying the gels/blots and plasmid maps.
