# Supplementary material for: Light control of the peptide-loading complex synchronizes antigen translocation and MHC I trafficking
Source: Commun Biol. 2021 Mar 30;4:430. doi: 10.1038/s42003-021-01890-z (PMC8010092; doi:10.1038/s42003-021-01890-z)
Supplement: Supplementary file 4 — Supplementary Data 1 [file 42003_2021_1890_MOESM4_ESM.pdf]

Figure 2 b

	core TAP		
	amber-free	amber mutation + BocK	amber mutation - BocK
mVenus positive cells (%)	25,60	6,50	0,16
	22,80	5,54	0,12
	21,70	5,33	0,14
	28,10	5,77	0,29
	27,60	5,67	0,21
	26,40	5,41	0,26
Mean (%)	25,37	5,70	0,20
SEM (%)	0,97	0,16	0,03
x-fold change		29,00	1,00

	TAP		
	amber-free	amber mutation + BocK	amber mutation - BocK
mVenus positive cells (%)	30,80	7,92	0,20
	31,50	7,31	0,20
	30,70	8,35	0,17
	18,60	4,58	0,14
	18,90	3,78	0,19
	19,90	3,70	0,23
Mean (%)	25,07	5,94	0,19
SEM (%)	2,43	0,80	0,01
x-fold change		31,54	1,00

Figure 3 a

	raw signal (a.u.)	normalized to amber-free coreTAP (%)
untr	92	0,33
coreTAP/optPylRS/PCK	27409	100,00
coreTAP(TAG)/optPylRS/PCK	8719	31,81
coreTAP(TAG)/optPylRS/-UAA	342	1,25
coreTAP(TAG)/optPylRS/BocK	334	1,22
coreTAP(TAG)/w/o PylRS/-UAA	500	1,83
coreTAP(TAG)/w/o PylRS/PCK	257	0,94
coreTAP(TAG)/wtPylRS/-UAA	692	2,53
coreTAP(TAG)/wtPylRS/BocK	11625	42,41
coreTAP(TAG)/wtPylRS/PCK	468	1,71

Figure 3 b

	coreTAP/PCK	coreTAP(TAG)/PCK
solubilisate TAP1	11817,288	6884,146
purified TAP1	8160,761	1137,719
purified TAP2mVenus	6580,669	900,506
norm to solubisate coreTAP/PCK		
solubilisate TAP1	1,00	0,583
purified TAP1	8160,76	1953,00
purified TAP2mVenus	6580,67	1545,804
set coreTAP/PCK to 100%		
TAP1 SBP elution	100,00	23,93
TAP2mVenus	100,00	23,49

Figure 3 c

	coreTAP/PCK	coreTAP(TAG)/PCK	coreTAP(TAG)/BocK
	7918	839	929
cells/single cells/mVenus-A subset |	7766	704	880
Mean (APC-Fire750-A) (a.u.)	8810	634	817
	8463	922	842
Mean (a.u.)	8239	775	867
SEM (a.u.)	210	56	21
set coreTAP/PCK to 100%			
	96,10	10,18	11,28
	94,26	8,54	10,68
single data points (%)	106,93	7,69	9,92
	102,72	11,19	10,22
Mean (%)	100,00	9,40	10,52
SEM (%)	3,60	0,72	0,37

Figure 4 b

	light-activated							
	coreTAP/PCK		coreTAP(TAG)/PCK		coreTAP(TAG)/BocK		mVenus/mock/PCK	
	ATP	ADP	ATP	ADP	ATP	ADP	ATP	ADP
cells/single cells/mVenus-A subset | Mean (Alexa Fluor 647-A) (a.u.)	59760	8219	16304	4677	6462	6038	3015	7104
	54147	7848	16538	6489	5528	6264	5054	6285
	54804	9849	21180	7393	3938	7740	3637	10507
Mean (a.u.)	56237	8639	18007	6186	5309	6681	3902	7965
SEM (a.u.)	1447	502	1296	652	602	436	493	1055
set light-activated coreTAP/PCK + ATP to 100%								
single data points (%)	106,26	14,61	28,99	8,32	11,49	10,74	5,36	12,63
	96,28	13,96	29,41	11,54	9,83	11,14	8,99	11,18
	97,45	17,51	37,66	13,15	7,00	13,76	6,47	18,68
Mean (%)	100,00	15,36	32,02	11,00	9,44	11,88	6,94	14,16
SEM (%)	3,64	0,98	2,45	1,19	1,10	0,83	0,89	1,91

Figure 4 c

	light-activated							
	TAP/PCK		TAP(TAG)/PCK		TAP(TAG)/BocK		mVenus/mock/PCK	
	ATP	ADP	ATP	ADP	ATP	ADP	ATP	ADP
cells/single cells/mVenus-A subset | Mean (Alexa Fluor 647-A) (a.u.)	54348	10797	33392	8359	6390	7197	6848	6886
	41590	7674	38228	7046	6183	6500	4947	7233
	48885	4872	37636	4093	3784	4924	3234	4373
Mean (a.u.)	48274	7781	36419	6499	5452	6207	5010	6164
SEM (a.u.)	3017	1397	1243	1030	683	549	852	736
set light-activated TAP/PCK + ATP to 100%								
single data points (%)	112,58	22,37	69,17	17,32	13,24	14,91	14,19	14,26
	86,15	15,90	79,19	14,60	12,81	13,46	10,25	14,98
	101,26	10,09	77,96	8,48	7,84	10,20	6,70	9,06
Mean (%)	100,00	16,12	75,44	13,46	11,29	12,86	10,38	12,77
SEM (%)	8,84	3,06	5,37	2,29	1,58	1,39	1,88	1,72

Figure 5 a, c

	coreTAP(TAG)/PCK						coreTAP/PCK	
	w/o hv	0 h	1 h	2 h	3 h	4 h	w/o hv	4 h
cells/single cells/mVenus-A subset | Mean (APC-Fire750-A) (a.u.)	885,0	850,0	1374,0	1686,0	1627,0	1870,0	6902,0	5749,0
	879,0	867,0	1369,0	1891,0	1952,0	1982,0	7592,0	6141,0
	1186,0	729,0	877,0	1193,0	1347,0	2037,0	7563,0	6834,0
Mean (a.u.)	983,3	815,3	1206,7	1590,0	1642,0	1963,0	7352,3	6241,3
SEM (a.u.)	82,8	35,5	134,6	169,1	142,7	40,1	184,0	259,0

One-way analysis of variance	
P value	< 0.0001
P value summary	***
Are means signif. different? (P < 0.05)	Yes
Number of groups	6
F	14,97
R squared	0,8618

ANOVA Table	SS	df	MS
Treatment (between columns)	2874000	5	574700
Residual (within columns)	460800	12	38400
Total	3334000	17	

Tukey's Multiple Comparison Test	Mean Diff.	q	Significant? P < 0.05?	Summary	95% CI of diff
w/o vs 0 h	168,0	1,485	No	ns	-369.5 to 705.5
w/o vs 1 h	-223,4	1,975	No	ns	-760.9 to 314.1
w/o vs 2 h	-606,7	5,363	Yes	*	-1144 to -69.19
w/o vs 3 h	-658,7	5,822	Yes	*	-1196 to -121.2
w/o vs 4 h	-979,7	8,659	Yes	***	-1517 to -442.2
0 h vs 1 h	-391,4	3,460	No	ns	-928.9 to 146.1
0 h vs 2 h	-774,7	6,847	Yes	**	-1312 to -237.2
0 h vs 3 h	-826,7	7,307	Yes	**	-1364 to -289.2
0 h vs 4 h	-1148	10,14	Yes	***	-1685 to -610.2
1 h vs 2 h	-383,3	3,388	No	ns	-920.8 to 154.2
1 h vs 3 h	-435,3	3,848	No	ns	-972.8 to 102.2
1 h vs 4 h	-756,3	6,685	Yes	**	-1294 to -218.8
2 h vs 3 h	-52,00	0,4596	No	ns	-589.5 to 485.5
2 h vs 4 h	-373,0	3,297	No	ns	-910.5 to 164.5
3 h vs 4 h	-321,0	2,837	No	ns	-858.5 to 216.5

Figure 5 b, d

	TAP(TAG)/PCK						TAP/PCK	
	w/o hv	0 h	1 h	2 h	3 h	4 h	w/o hv	4 h
cells/single cells/mVenus-A subset | Mean (APC-Fire750-A) (a.u.)	1241,0	1195,0	1748,0	2128,0	2508,0	2598,0	7512,0	8275,0
	1933,0	1385,0	1543,0	1978,0	2387,0	2456,0	7407,0	7356,0
	1355,0	1243,0	1927,0	2198,0	2411,0	2640,0	7482,0	10248,0
Mean (a.u.)	1509,7	1274,3	1739,3	2101,3	2435,3	2564,7	7467,0	8626,3
SEM (a.u.)	174,9	46,6	90,6	53,0	30,2	45,5	25,5	696,6

One-way analysis of variance	
P value	< 0.0001
P value summary	***
Are means signif. different? (P < 0.05)	Yes
Number of groups	6
F	34,13
R squared	0,9343

ANOVA Table	SS	df	MS
Treatment (between columns)	3990000	5	798100
Residual (within columns)	280600	12	23380
Total	4271000	17	

Tukey's Multiple Comparison Test	Mean Diff.	q	Significant? P < 0.05?	Summary	95% CI of diff
w/o vs 0 h	235,4	2,666	No	ns	-184.0 to 654.8
w/o vs 1 h	-229,6	2,601	No	ns	-649.0 to 189.8
w/o vs 2 h	-591,6	6,701	Yes	**	-1011 to -172.2
w/o vs 3 h	-925,6	10,48	Yes	***	-1345 to -506.2
w/o vs 4 h	-1055	11,95	Yes	***	-1474 to -635.6
0 h vs 1 h	-465,0	5,267	Yes	*	-884.4 to -45.58
0 h vs 2 h	-827,0	9,368	Yes	***	-1246 to -407.6
0 h vs 3 h	-1161	13,15	Yes	***	-1580 to -741.6
0 h vs 4 h	-1290	14,62	Yes	***	-1710 to -871.0
1 h vs 2 h	-362,0	4,101	No	ns	-781.4 to 57.42
1 h vs 3 h	-696,0	7,884	Yes	**	-1115 to -276.6
1 h vs 4 h	-825,4	9,350	Yes	***	-1245 to -406.0
2 h vs 3 h	-334,0	3,783	No	ns	-753.4 to 85.42
2 h vs 4 h	-463,4	5,249	Yes	*	-882.8 to -43.98
3 h vs 4 h	-129,4	1,466	No	ns	-548.8 to 290.0

Supplementary Fig. 2

	PCK (nM)								
	0	1	2	3	4	5	10	15	20
cells/single cells/mVenus-A subset | Mean (mVenus-A) (a.u.)	1869,0	1680,0	1879,0	2020,0	1902,0	2199,0	2340,0	2083,0	2293
	1785,0	1813,0	1893,0	2034,0	2087,0	2075,0	2152,0	2201,0	2353
	1722,0	1516,0	1960,0	1889,0	2028,0	2110,0	2259,0	2342,0	2342
Mean (a.u.)	1792,0	1669,7	1910,7	1981,0	2005,7	2128,0	2250,3	2208,7	2329,3
SEM (a.u.)	34,8	70,1	20,4	37,7	44,5	30,1	44,5	61,1	15,1

	PCK (nM)					
	30	40	50	100	250	500
cells/single cells/mVenus-A subset | Mean (mVenus-A) (a.u.)	2395	2417	2360	2577	2524	2434
	2521	2452	2558	2524	2557	2438
	2384	2494	2423	2581	2662	2455
Mean (a.u.)	2433,3	2454,3	2447,0	2560,7	2581,0	2442,3
SEM (a.u.)	35,9	18,2	47,7	15,0	34,0	5,3

Supplementary Fig. 4 a

	w/o illumination							
	coreTAP/PCK		coreTAP(TAG)/PCK		coreTAP(TAG)/BocK		mVenus/mock/PCK	
	ATP	ADP	ATP	ADP	ATP	ADP	ATP	ADP
cells/single cells/mVenus-A subset | Mean (Alexa Fluor 647-A) (a.u.)	19132	6039	3632	5650	3816	5276	3470	6484
	62202	8934	5890	7837	6006	7606	3694	8376
	76204	7951	6900	6508	6408	7132	6380	8014
Mean (a.u.)	52513	7641	5474	6665	5410	6671	4515	7625
SEM (a.u.)	14022	694	789	519	658	580	763	473
set light-activated coreTAP/PCK + ATP to 100%								
single data points (%)	34,02	10,74	6,46	10,05	6,79	9,38	6,17	11,53
	110,61	15,89	10,47	13,94	10,68	13,52	6,57	14,89
	135,51	14,14	12,27	11,57	11,39	12,68	11,34	14,25
Mean (%)	93,38	13,59	9,73	11,85	9,62	11,86	8,03	13,56
SEM (%)	25,05	1,28	1,42	0,97	1,20	1,08	1,37	0,91

	light-activated							
	coreTAP/PCK		coreTAP(TAG)/PCK		coreTAP(TAG)/BocK		mVenus/mock/PCK	
	ATP	ADP	ATP	ADP	ATP	ADP	ATP	ADP
cells/single cells/mVenus-A subset | Mean (Alexa Fluor 647-A) (a.u.)	59760	8219	16304	4677	6462	6038	3015	7104
	54147	7848	16538	6489	5528	6264	5054	6285
	54804	9849	21180	7393	3938	7740	3637	10507
Mean (a.u.)	56237	8639	18007	6186	5309	6681	3902	7965
SEM (a.u.)	1447	502	1296	652	602	436	493	1055
set light-activated coreTAP/PCK + ATP to 100%								
single data points (%)	106,26	14,61	28,99	8,32	11,49	10,74	5,36	12,63
	96,28	13,96	29,41	11,54	9,83	11,14	8,99	11,18
	97,45	17,51	37,66	13,15	7,00	13,76	6,47	18,68
Mean (%)	100,00	15,36	32,02	11,00	9,44	11,88	6,94	14,16
SEM (%)	3,64	0,98	2,45	1,19	1,10	0,83	0,89	1,91

One-way analysis of variance	coreTAP(TAG)/PCK
P value	< 0.0001
P value summary	***
Are means signif. different? (P < 0.05)	Yes
Number of groups	4
F	43,45
R squared	0,9422

ANOVA Table	SS	df	MS
Treatment (between columns)	1014	3	338,1
Residual (within columns)	62,26	8	7,782
Total	1077	11	

Tukey's Multiple Comparison Test	Mean Diff.	q	Significant? P < 0.05?	Summary	95% CI of diff
ADP vs ADP + hv	0,8500	0,5278	No	ns	-6.444 to 8.144
ADP vs ATP	2,120	1,316	No	ns	-5.174 to 9.414
ADP vs ATP +hv	-20,17	12,52	Yes	***	-27.46 to -12.88
ADP + hv vs ATP	1,270	0,7885	No	ns	-6.024 to 8.564
ADP + hv vs ATP +hv	-21,02	13,05	Yes	***	-28.31 to -13.73
ATP vs ATP +hv	-22,29	13,84	Yes	***	-29.58 to -15.00

Supplementary Fig. 4 b

	w/o illumination							
	TAP/PCK		TAP(TAG)/PCK		TAP(TAG)/BocK		mVenus/mock/PCK	
	ATP	ADP	ATP	ADP	ATP	ADP	ATP	ADP
cells/single cells/mVenus-A subset | Mean (Alexa Fluor 647-A) (a.u.)	53355	9694	7791	9418	7898	11529	5958	11118
	57877	7544	6128	6357	5814	6456	5432	6849
	57054	5508	3681	4684	3680	3972	3102	4364
Mean (a.u.)	56095	7582	5867	6820	5797	7319	4831	7444
SEM (a.u.)	1135	987	975	1132	994	1816	717	1610
set light-activated TAP/PCK + ATP to 100%								
	110,52	20,08	16,14	19,51	16,36	23,88	12,34	23,03
single data points (%)	119,89	15,63	12,69	13,17	12,04	13,37	11,25	14,19
	118,19	11,41	7,63	9,70	7,62	8,23	6,43	9,04
Mean (%)	116,20	15,71	12,15	14,13	12,01	15,16	10,01	15,42
SEM (%)	7,63	2,27	2,16	2,51	2,19	3,88	1,61	3,47

	light-activated							
	TAP/PCK		TAP(TAG)/PCK		TAP(TAG)/BocK		mVenus/mock/PCK	
	ATP	ADP	ATP	ADP	ATP	ADP	ATP	ADP
cells/single cells/mVenus-A subset | Mean (Alexa Fluor 647-A) (a.u.)	54348	10797	33392	8359	6390	7197	6848	6886
	41590	7674	38228	7046	6183	6500	4947	7233
	48885	4872	37636	4093	3784	4924	3234	4373
Mean (a.u.)	48274	7781	36419	6499	5452	6207	5010	6164
SEM (a.u.)	3017	1397	1243	1030	683	549	852	736
set light-activated TAP/PCK + ATP to 100%								
	112,58	22,37	69,17	17,32	13,24	14,91	14,19	14,26
single data points (%)	86,15	15,90	79,19	14,60	12,81	13,46	10,25	14,98
	101,26	10,09	77,96	8,48	7,84	10,20	6,70	9,06
Mean (%)	100,00	16,12	75,44	13,46	11,29	12,86	10,38	12,77
SEM (%)	8,84	3,06	5,37	2,29	1,58	1,39	1,88	1,72

One-way analysis of variance	TAP(TAG)/PCK
P value	< 0.0001
P value summary	***
Are means signif. different? (P < 0.05)	Yes
Number of groups	4
F	85,90
R squared	0,9699

ANOVA Table	SS	df	MS
Treatment (between columns)	8709	3	2903
Residual (within columns)	270,4	8	33,79
Total	8979	11	

Tukey's Multiple Comparison Test	Mean Diff.	q	Significant? P < 0.05?	Summary	95% CI of diff
ADP vs ADP +hv	0,6636	0,1977	No	ns	-14.54 to 15.86
ADP vs ATP	1,974	0,5882	No	ns	-13.23 to 17.17
ADP vs ATP +hv	-61,31	18,27	Yes	***	-76.51 to -46.11
ADP +hv vs ATP	1,311	0,3905	No	ns	-13.89 to 16.51
ADP +hv vs ATP +hv	-61,98	18,47	Yes	***	-77.18 to -46.78
ATP vs ATP +hv	-63,29	18,86	Yes	***	-78.49 to -48.09

Supplementary Fig. 5

	coreTAP				
	0 h	2 h	4 h	6 h	8 h
cells/single cells/mVenus-A subset | Mean (APC-Fire750-A) (a.u.)	1689	3952	5933	7205	7637
	1439	3466	5487	6939	6712
	1676	3934	6280	6903	7730
Mean (a.u.)	1601,3	3784,0	5900,0	7015,7	7359,7
SEM (a.u.)	66,3	129,9	187,4	77,8	265,3
