# Supplementary material for: Light control of the peptide-loading complex synchronizes antigen translocation and MHC I trafficking
Source: Commun Biol. 2021 Mar 30;4:430. doi: 10.1038/s42003-021-01890-z (PMC8010092; doi:10.1038/s42003-021-01890-z)
Supplement: Supplementary file 5 — Supplementary Data 2 [file 42003_2021_1890_MOESM5_ESM.pdf]

Plasmid maps

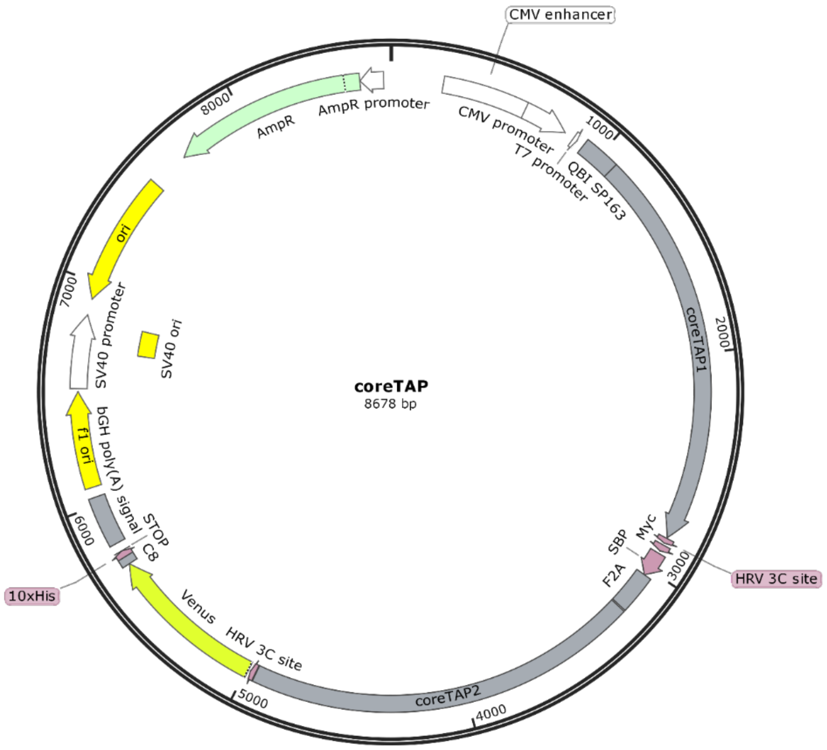

Plasmid encoding for coreTAP

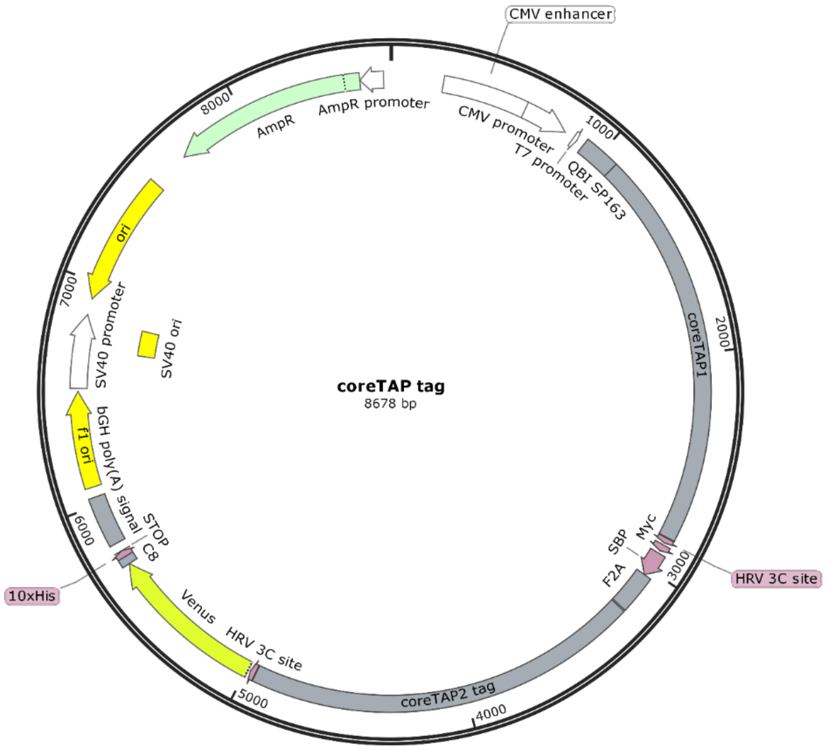

Plasmid encoding for coreTAP^{TAG}

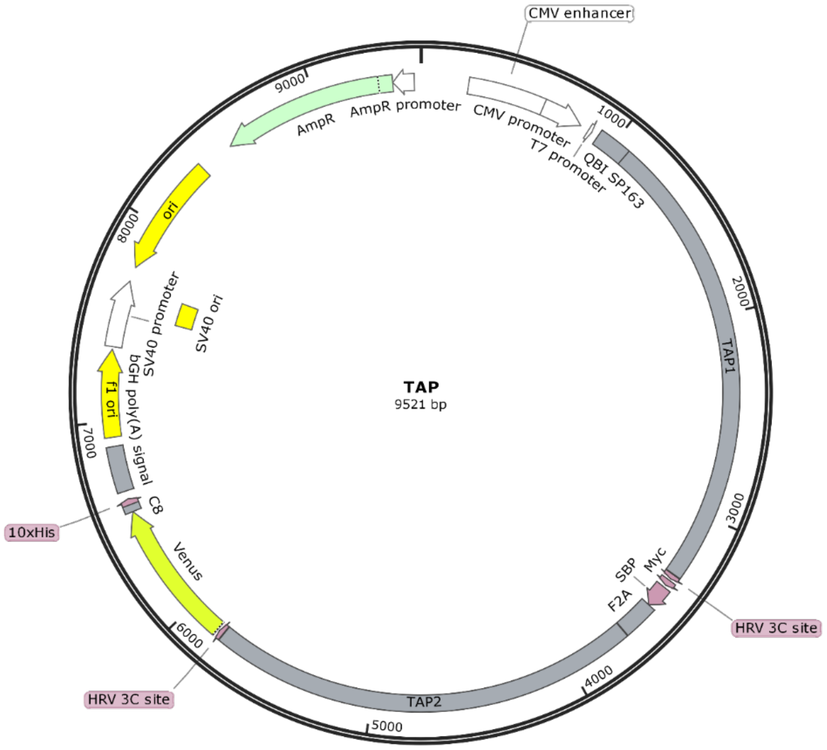

Plasmid encoding for TAP

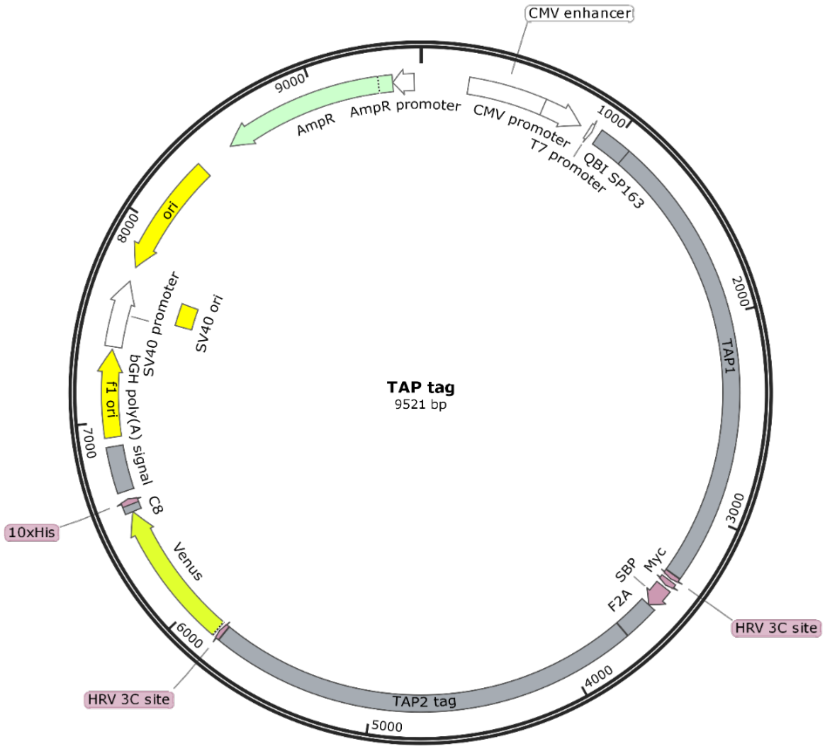

Plasmid encoding for TAP^{TAG}

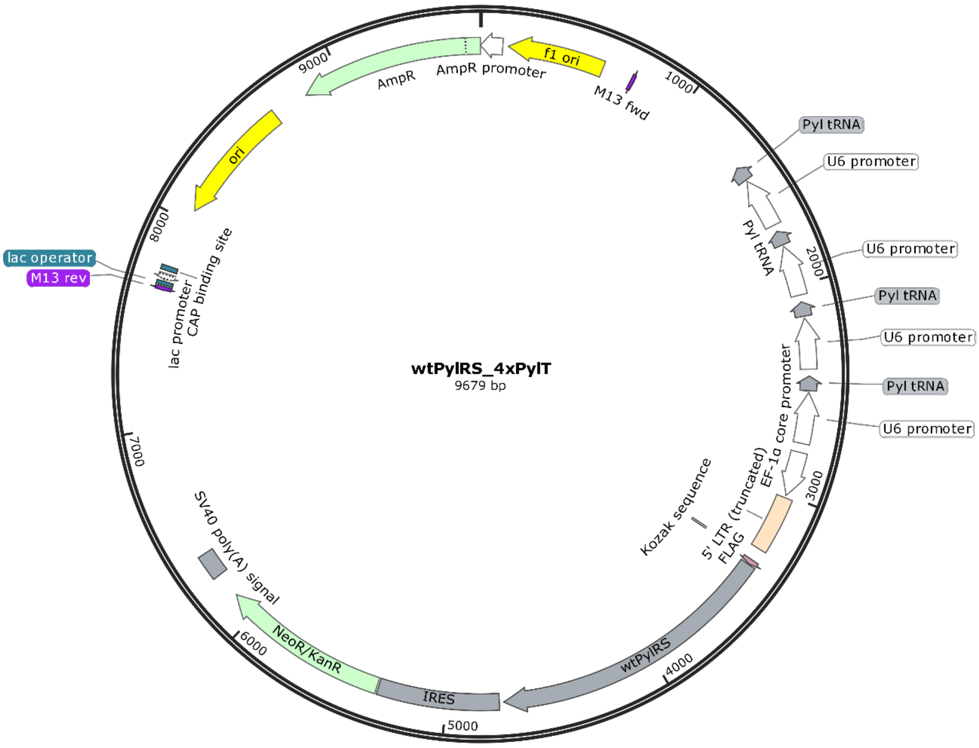

Plasmid encoding for wtPylRS

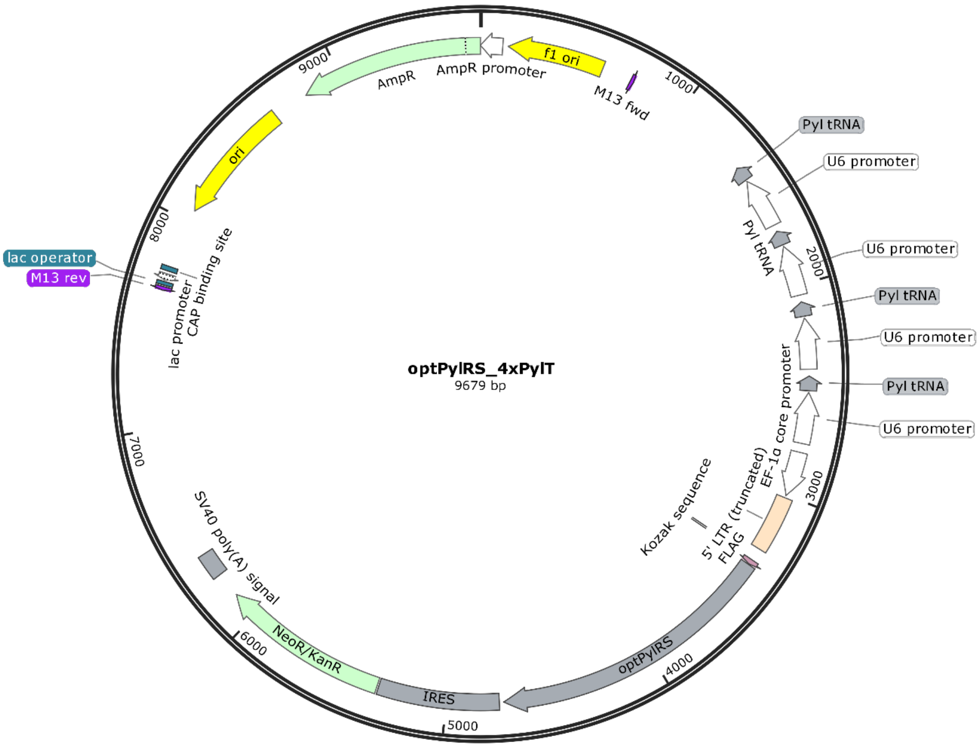

Plasmid encoding for optPylRS

Figure 2 c

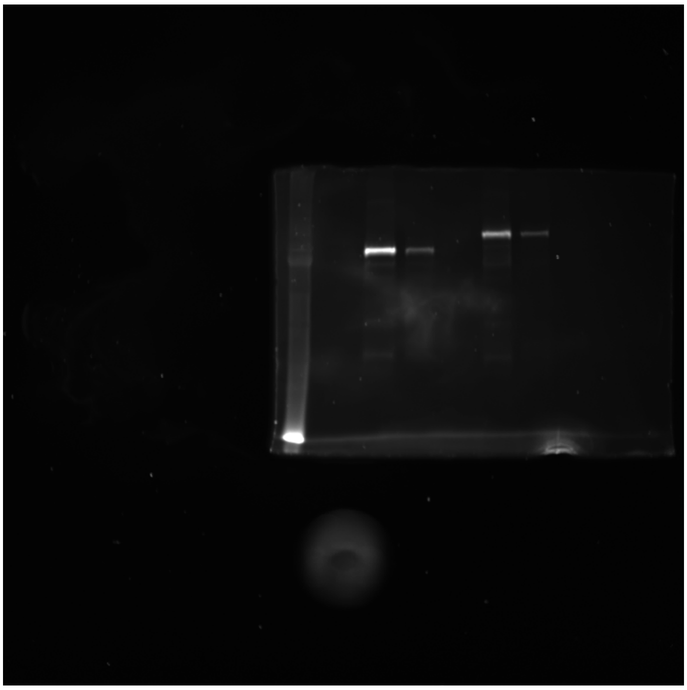

mVenus in-gel fluorescence (ex/em 480/535)

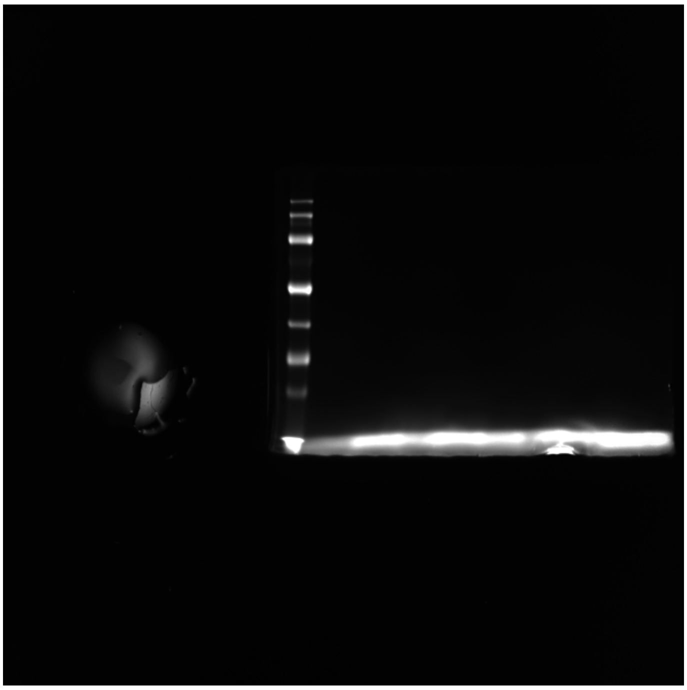

mVenus in-gel fluorescence marker image

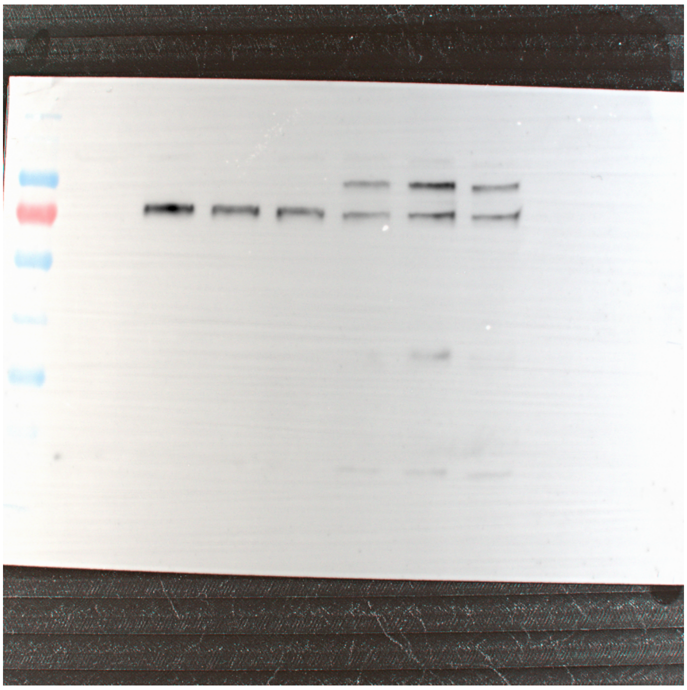

anti-TAP1 (148.3)

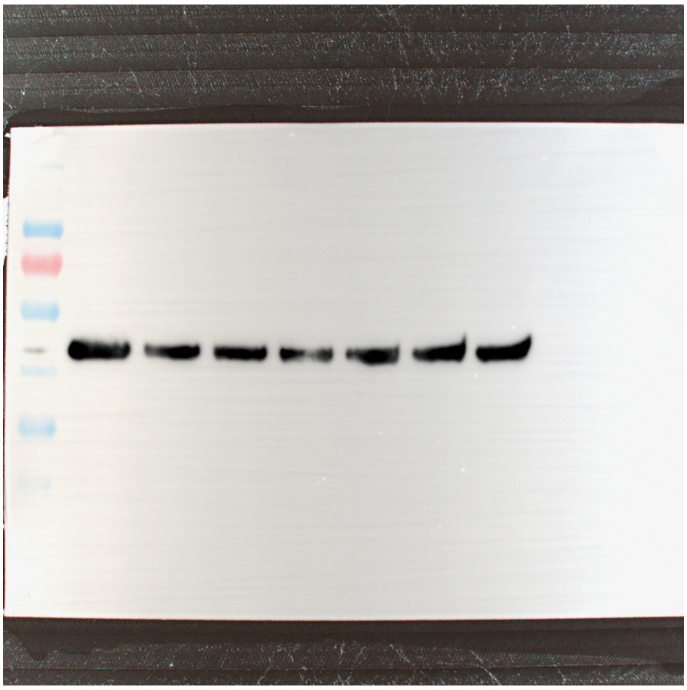

anti-β-actin (AC-74, Sigma-Aldrich)

Figure 3 a

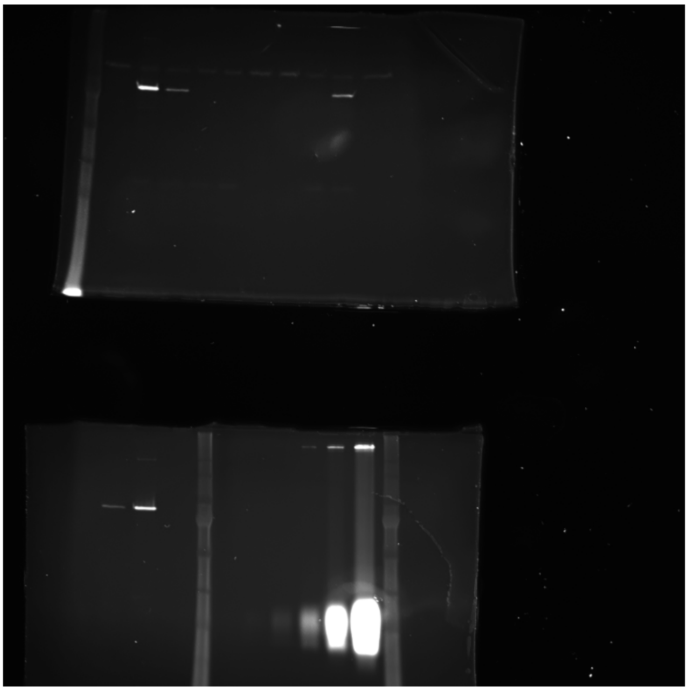

mVenus in-gel fluorescence (ex/em 480/535)

(upper gel)

Figure 3 b

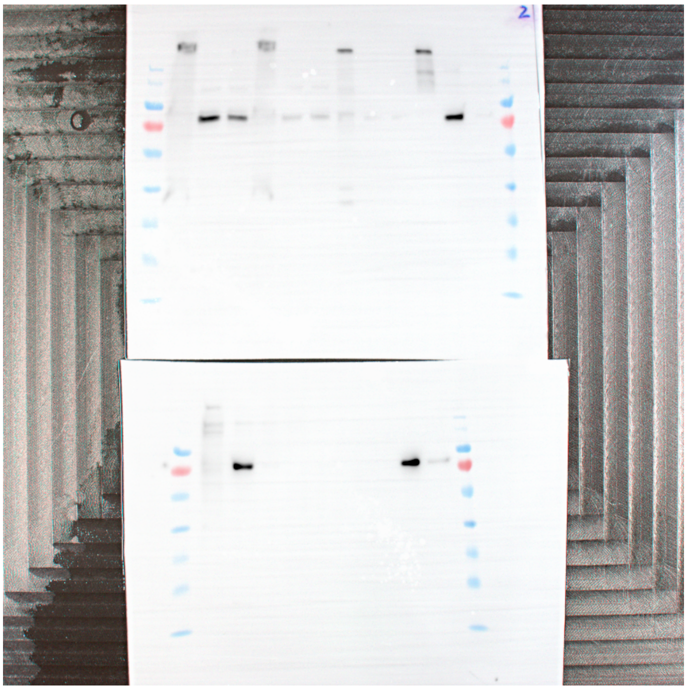

input, anti-TAP1 (148.3)

(upper blot, lane 1-4 from the left)

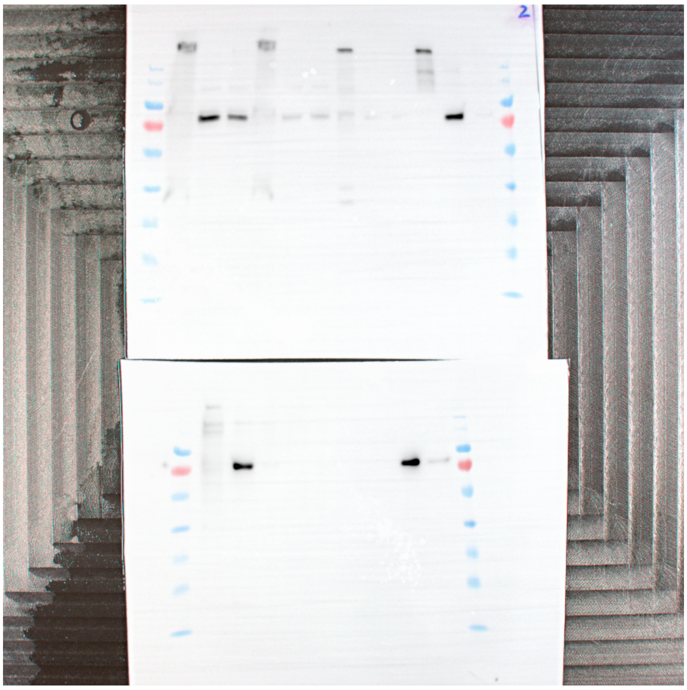

purified protein, anti-TAP1 (148.3)

(lower blot, lane 1-4 from the right)

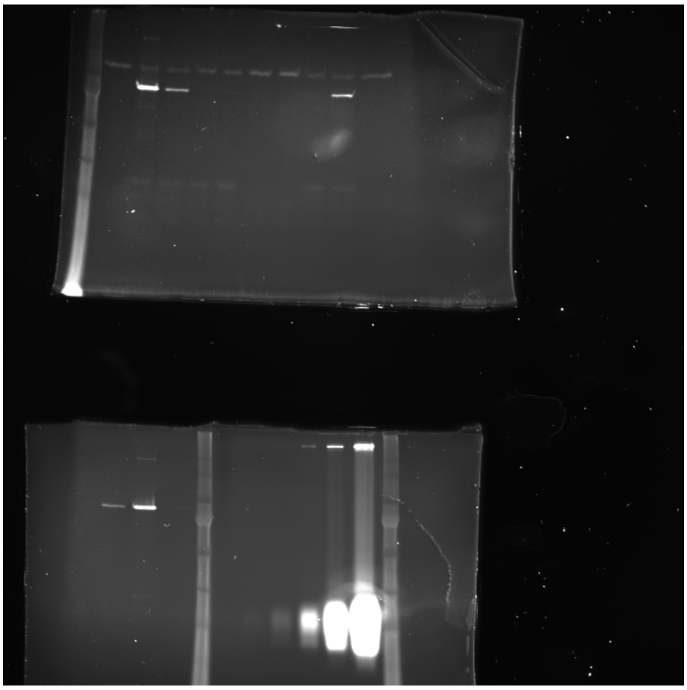

purified protein, mVenus in-gel fluorescence (ex/em 480/535)

(lower gel, lane 1-4 from the left)
