# Supplementary material for: Light control of the peptide-loading complex synchronizes antigen translocation and MHC I trafficking
Source: Commun Biol. 2021 Mar 30;4:430. doi: 10.1038/s42003-021-01890-z (PMC8010092; doi:10.1038/s42003-021-01890-z)
Supplement: Supplementary file 6 — Reporting Summary [file 42003_2021_1890_MOESM6_ESM.pdf]

nature research

Corresponding author(s): Robert Tampé

Last updated by author(s): Feb 4, 2021

Reporting Summary

Nature Research wishes to improve the reproducibility of the work that we publish. This form provides structure for consistency and transparency in reporting. For further information on Nature Research policies, see our Editorial Policies and the Editorial Policy Checklist.

Statistics

For all statistical analyses, confirm that the following items are present in the figure legend, table legend, main text, or Methods section.

n/a	Confirmed	
☐	☒	The exact sample size (n) for each experimental group/condition, given as a discrete number and unit of measurement
☐	☒	A statement on whether measurements were taken from distinct samples or whether the same sample was measured repeatedly
☐	☒	The statistical test(s) used AND whether they are one- or two-sided<br>*Only common tests should be described solely by name; describe more complex techniques in the Methods section.*
☒	☐	A description of all covariates tested
☒	☐	A description of any assumptions or corrections, such as tests of normality and adjustment for multiple comparisons
☐	☒	A full description of the statistical parameters including central tendency (e.g. means) or other basic estimates (e.g. regression coefficient) AND variation (e.g. standard deviation) or associated estimates of uncertainty (e.g. confidence intervals)
☐	☒	For null hypothesis testing, the test statistic (e.g. F, t, r) with confidence intervals, effect sizes, degrees of freedom and P value noted<br>*Give P values as exact values whenever suitable.*
☒	☐	For Bayesian analysis, information on the choice of priors and Markov chain Monte Carlo settings
☒	☐	For hierarchical and complex designs, identification of the appropriate level for tests and full reporting of outcomes
☒	☐	Estimates of effect sizes (e.g. Cohen's d, Pearson's r), indicating how they were calculated

Our web collection on statistics for biologists contains articles on many of the points above.

Software and code

Policy information about availability of computer code

Data collection: n.a.

Data analysis: n.a.

For manuscripts utilizing custom algorithms or software that are central to the research but not yet described in published literature, software must be made available to editors and reviewers. We strongly encourage code deposition in a community repository (e.g. GitHub). See the Nature Research guidelines for submitting code & software for further information.

Data

Policy information about availability of data

All manuscripts must include a data availability statement. This statement should provide the following information, where applicable:

- Accession codes, unique identifiers, or web links for publicly available datasets
- A list of figures that have associated raw data
- A description of any restrictions on data availability

All data are accessible from the corresponding author upon reasonable request. The raw data underlying any graph is provided as a source data file.

Field-specific reporting

Please select the one below that is the best fit for your research. If you are not sure, read the appropriate sections before making your selection.

☒ Life sciences ☐ Behavioural & social sciences ☐ Ecological, evolutionary & environmental sciences

For a reference copy of the document with all sections, see nature.com/documents/nr-reporting-summary-flat.pdf

Life sciences study design

All studies must disclose on these points even when the disclosure is negative.

Sample size	n.a.
Data exclusions	no data were excluded from the analysis
Replication	all attempts of replication were successful
Randomization	n.a.
Blinding	n.a.

Reporting for specific materials, systems and methods

We require information from authors about some types of materials, experimental systems and methods used in many studies. Here, indicate whether each material, system or method listed is relevant to your study. If you are not sure if a list item applies to your research, read the appropriate section before selecting a response.

Materials & experimental systems

n/a	Involved in the study	
☐	☒	Antibodies
☐	☒	Eukaryotic cell lines
☒	☐	Palaeontology and archaeology
☒	☐	Animals and other organisms
☒	☐	Human research participants
☒	☐	Clinical data
☒	☐	Dual use research of concern

Methods

n/a	Involved in the study	
☒	☐	ChIP-seq
☐	☒	Flow cytometry
☒	☐	MRI-based neuroimaging

Antibodies

Antibodies used	anti-TAP1 148.3, hybridoma supernatant, mouse, generated in-house (Meyer et al. 1994 FEBS Lett. 351(3):443-7)<br>anti-β-actin antibody, monoclonal, mouse, Sigma-Aldrich (clone AC-74, Cat # A2228)<br>anti-HLA-A,B,C antibody, mouse IgG2a, labeled with APC/Fire™ 750, Bio-Legend (clone W6/32, Cat # 311444)
Validation	anti-β-actin antibody is validated for immunoblotting of human actin following manufacturer website<br>anti-HLA-A,B,C antibody is validated for flow cytometry of human MHC class I following manufacturer website

Eukaryotic cell lines

Policy information about cell lines

Cell line source(s)	STF1-169, adherent TAP2-deficient fibroblast cells, provided by Henry de la Salle, University of Strasbourg, France<br>FreeStyle™ 293 F cells, human embryonic kidney cells adapted to suspension growth, Gibco™ (Cat # R79007)
Authentication	n.a.
Mycoplasma contamination	all cells used were tested mycoplasm negative
Commonly misidentified lines<br>(See ICLAC register)	n.a.

Flow Cytometry

Plots

Confirm that:

☒ The axis labels state the marker and fluorochrome used (e.g. CD4-FITC).

☒ The axis scales are clearly visible. Include numbers along axes only for bottom left plot of group (a 'group' is an analysis of identical markers).

☒ All plots are contour plots with outliers or pseudocolor plots.

☒ A numerical value for number of cells or percentage (with statistics) is provided.

Methodology

Sample preparation	Cells were detached by using trypsin, washed with FACS-buffer (2% FCS in PBS), blocked with 5% BSA for 15 min, washed and stained with 1.5% APC-Fire750 anti-human HLA-A,B,C for 20 min, washed and resupended for analysis. Staining procedure was performed at 4 °C.
Instrument	BD FACSCelesta, Blue, Violet, and Red Laser Base Configuration
Software	FlowJo V10 software
Cell population abundance	Abundance of construct expressing cells was determined by gating on the mVenus positive cells using FlowJo V10 software. Depending on the plasmid 5-55% of the parental gate were construct expressing cells.
Gating strategy	For detection of surface MHC I, cell population was gated based on size and granularity (SSC-A/FSC-A), duplet discrimination was performed (FSC-H/FSC-A) and construct expressing cells were gated based on mVenus fluorescence (dot plot or histogram).<br>For peptide transport, cell population was gated based on size and granularity (SSC-A/FSC-A) and construct expressing cells were gated based on mVenus fluorescence (dot plot or histogram).

☒ Tick this box to confirm that a figure exemplifying the gating strategy is provided in the Supplementary Information.
